# A pre-clinical PET scanner based on a monolithic annulus of scintillator (AnnPET): construction and NU4-2008 performance testing

**DOI:** 10.1088/1361-6560/adc537

**Published:** 2025-04-08

**Authors:** Raymond R Raylman, Alexander V Stolin, Gangadhar Jaliparthi, Peter F Martone

**Affiliations:** Center for Advanced Imaging, Department of Radiology, One Medical Center Dr, Box 9236, West Virginia University, Morgantown, WV 26506, United States of America

**Keywords:** positron emission tomography, monolithic scintillator, pre-clinical

## Abstract

*Objective.* In the past several decades, numerous positron emission tomography (PET) scanners of various designs have been constructed for use in pre-clinical studies. Our group is investigating use of a monolithic annulus of scintillator, instead of the traditional arrays of discrete scintillator elements or individual detectors that utilize continuous blocks of scintillator, to construct a novel pre-clinical PET scanner. *Approach.* This scanner, called AnnPET, is based on a fourteen-faceted annulus of lutetium yttrium orthosilicate with an inner diameter of 6 cm and length of 7.2 cm. Each facet is populated with four specially constructed 4 × 4 arrays of 4 mm × 4 mm multi-pixel photon counters .To cool and temperature stabilize these devices, the scanner gantry is immersed in dielectric fluid. Positioning of events in the scintillator is accomplished with the application of deep-residual convolutional neural network. The scanner’s performance was assessed using the NEMA NU4-2008 protocols. *Results.* Full-width-at-half-maximum (FWHM) of the images of a point source reconstructed with the single slice rebinned filtered backprojection (SSRB-FBP) algorithm at 5 mm from the center of the scanner are: 1.40 mm (radial), 1.38 mm (tangential) and 1.40 mm (axial). At 18 mm from scanner center (edge of the scanner’s inner bore) the FWHMs are: 1.62 mm (radial), 1.43 mm (tangential) and 1.48 mm (axial) FWHM. Peak detection sensitivity is 9.5% (0.086 cps Bq^−1^). Peak noise equivalent count rate is 234 kcps at 14.4 MBq. *Significance.* Overall, testing of the AnnPET system demonstrated very promising performance results for a pre-clinical PET scanner based on a single, cooled annulus of monolithic scintillator used with neural networks. Continued development of the system is planned.

## 1. Introduction

Development of pre-clinical positron emission tomography (PET) scanners has transformed how small animals are used in research (Judenhofer and Cherry 2013, Miyaoka and Lehnert 2020). These systems facilitate longitudinal investigations of large groups of animals to study cancer and other diseases. To accommodate the anatomy of small animals (often rats or mice), they are required to possess high spatial resolution and detection sensitivity (Goertzen *et al* 2012, Yamamoto *et al* 2013, Yang *et al* 2016, Kang *et al* 2021).

Often high spatial resolution is achieved by utilizing arrays of very small discrete scintillator elements to localize interaction points of annihilation photons in the scanner. These points represent the two ends of a line-of-response (LOR), defining the path of the annihilation photons through the object. The spatial resolution of a PET scanner is largely dependent upon by how accurately these interaction points are determined, given the physical effects of positron range and annihilation photon noncollinearity on resolution. For PET scanners that utilizes pixelated detectors, spatial resolution is limited by the cross-sectional extent of the scintillator elements that make up the detector and the physical phenomena noted above. Additionally, the radial component of spatial resolution is affected by depth-of-interaction (DOI), which degrades the ability to accurately determine the radial position component of the LOR endpoints in discrete detector elements. Use of continuous scintillator-based detectors promise more accurate estimates of LOR endpoints, enhancing spatial resolution, by utilizing shape of the optical photon distribution to estimate event depth (Ling *et al* 2007, Wang *et al* 2013). Several scanners have been constructed that utilize rings of individual detector modules based on monolithic blocks of scintillator (Krishnamoorthy *et al* 2018, Freire *et al* 2022). One of the weaknesses of this design is that the gaps between parallelepiped detectors diminish detection sensitivity. Additionally, monolithic scintillator-based detectors often require sophisticated methods to accurately identify the three-dimensional interaction points in the scintillator (Ling *et al* 2007, Wang *et al* 2013).

To address the necessity to simultaneously achieve high spatial resolution and high detection sensitivity in pre-clinical PET scanners, we introduced the concept of a scanner based on a single, continuous, faceted, annulus of scintillator, called AnnPET in 2016 (Stolin *et al* 2016). The current version of AnnPET utilizes a tetradecagonal annulus of cerium doped lutetium yttrium orthosilicate (LYSO) coupled to arrays of multi-pixel photon counters (MPPC) cooled in a tank of dielectric fluid (Stolin *et al* 2017, Jaliparthi *et al* 2021). In addition to potential gains in performance, PET scanners based on single annuli of monolithic scintillator are less complex and less expensive to construct than those that utilize rings of detectors that consist of arrays of discrete elements or monolithic blocks of scintillator. Specifically, the detector modules in these systems ware relatively expensive to fabricate and the support structures to mount them are relatively complex.

Recently, two groups have investigated pre-clinical PET scanners utilizing continuous annuli of scintillator (a head only clinical PET scanner based on a continuous annulus of sodium iodide was constructed in 1997 Karp *et al* (1997)). Specifically, a group from the Huazhong University of Science and Technology in Wuhan China constructed a PET scanner based on a 25.1 mm long LYSO cylinder with an inner diameter of 48.5 mm and outer diameter of 58.5 mm (Xu *et al* 2019). Scintillation light was detected at each end of the cylinder with rings of 3 mm × 3 mm silicon photomultipliers (SiPM). Outputs from these devices were used to measure the circumferential angle and axial position of each annihilation event. Additionally, the i3M group in Valencia Spain constructed a scanner utilizing a monolithic annulus of LYSO with ten facets machined into its surface (Freire *et al* 2022). They chose to populate only a fraction (approximately one third) of each facet with 8 × 8 arrays of 3 mm × 3 mm SiPMs. In this work we describe the construction of AnnPET and assessment of its performance using the NEMA NU4-2008 protocol (NEMA, National Electrical Manufacturers Association 2008).

## 2. Methods

### 2.1. Detector design and construction

**Figure 1.**
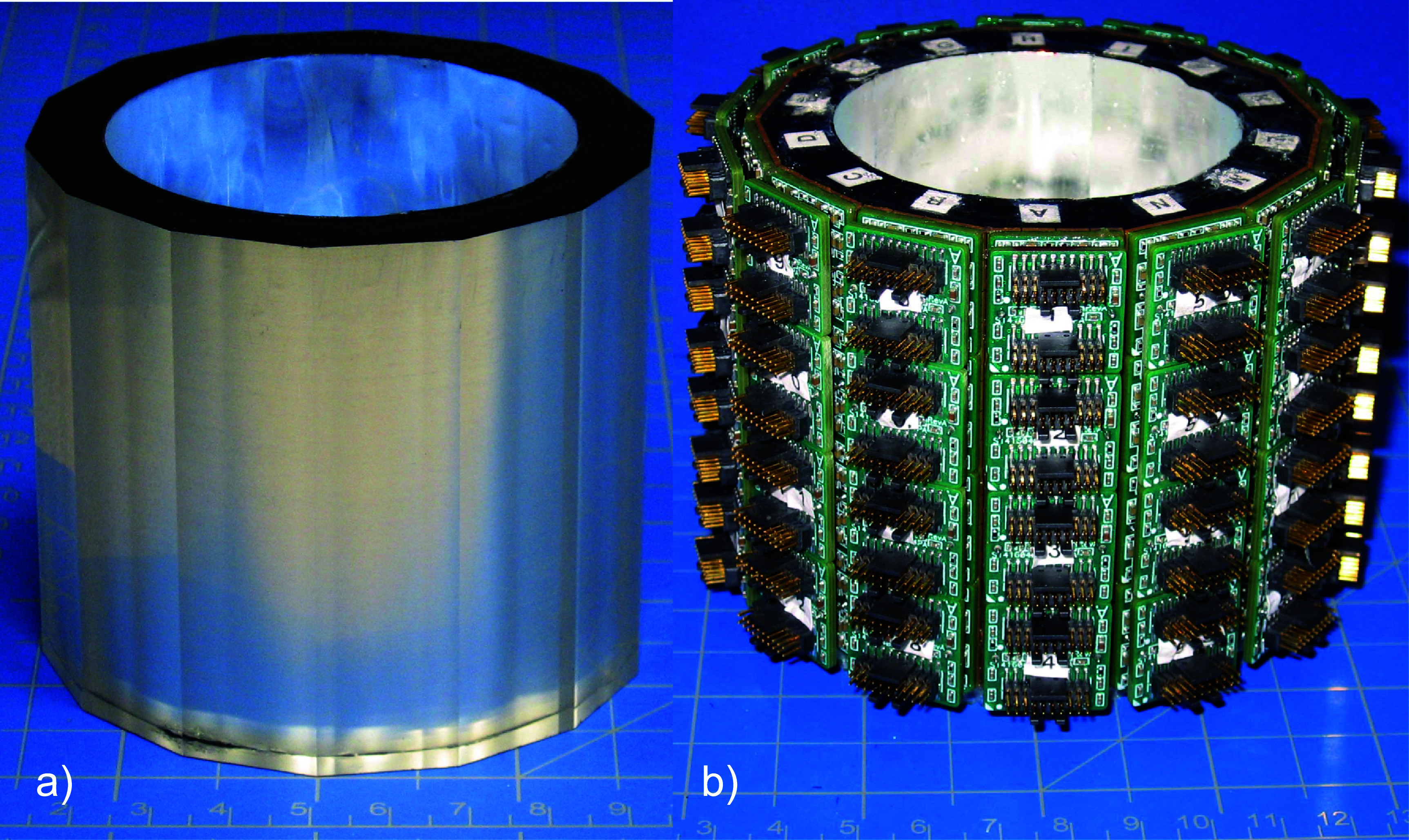
Annular AnnPET detector. (a) LYSO annulus and (b) annulus with its surface populated with MPPC arrays/readout electronics.

AnnPET’s monolithic LYSO scintillator annulus is 7.2 cm long with a maximum outer diameter of 8.2 cm and inner diameter of 6.0 cm (appropriate for imaging of mice) (Proteus, Inc., Chagrin Falls, OH). Fourteen 18.2 mm-wide facets were machined equidistantly around the outer surface of the annulus. The minimum thickness of scintillator is 9.7 mm at the center of a facet. The inner surface was covered with a specular reflector (3M Corp., Maplewood, MN) to maximize collection of the optical photons produced by interaction of annihilation photons in the scintillator. Each end is coated with black absorptive paint to reduce reflection of scintillation photons from these surfaces. Figure 1(a) shows the annulus. Scintillation photons were detected by custom fabricated, 4 × 4 arrays of 4 mm × 4 mm (pitch = 4.4 mm) S14160/S14161-4050HS MPPC (Hamamatsu Photonics, KK, Hamamatsu City, Japan) constructed by AiT Technologies, LLC (Newport News, VA). The sixteen MPPCs in each array were gain matched to facilitate uniformity in the measurements of scintillation photon distributions. Four MPPC arrays were bonded to each facet using an optically transparent silastic (Sylgard 184, Dow Chemical Corp., Midland, MI). Figure 1(b) shows the annulus with MPPC arrays attached.

### 2.2. Scanner gantry

**Figure 2.**
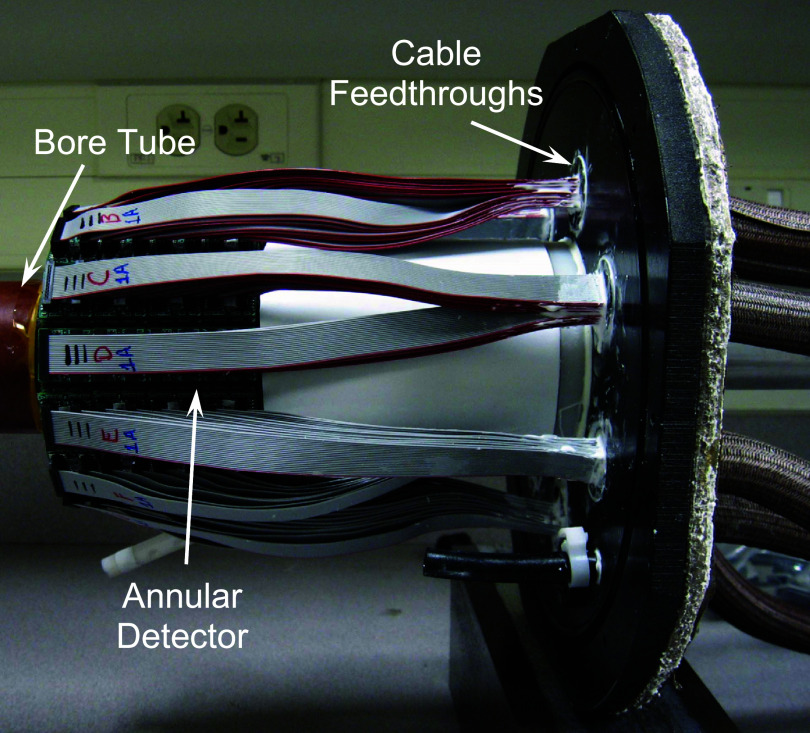
Detector mounted on the central bore tube. The detector cables are shown passing through the rear endplate.

The scanner gantry was constructed using non-magnetic materials in anticipation of combining AnnPET with an electron paramagnetic resonance imaging (EPRI) system (Tseytlin *et al* 2018). The detector, shown in figure 1(b), was mounted on a Garolite ^TM^ cylinder (outer diameter = 6 cm, inner diameter = 5 cm). This tube passes through the center of the annulus and each endplate that constitute the main support structures of the scanner. The cylinder’s penetrations in the endplates are sealed using dual, internal O-rings to ensure that the gantry is fluid-tight since it will be filled with dielectric fluid. The ribbon cables that connect the MPPC readout electronics to their control modules pass through the rear endplate using fluid-tight feedthroughs fabricated from 1.25 cm-thick Delrin ^TM^ (figure 2).

**Figure 3.**
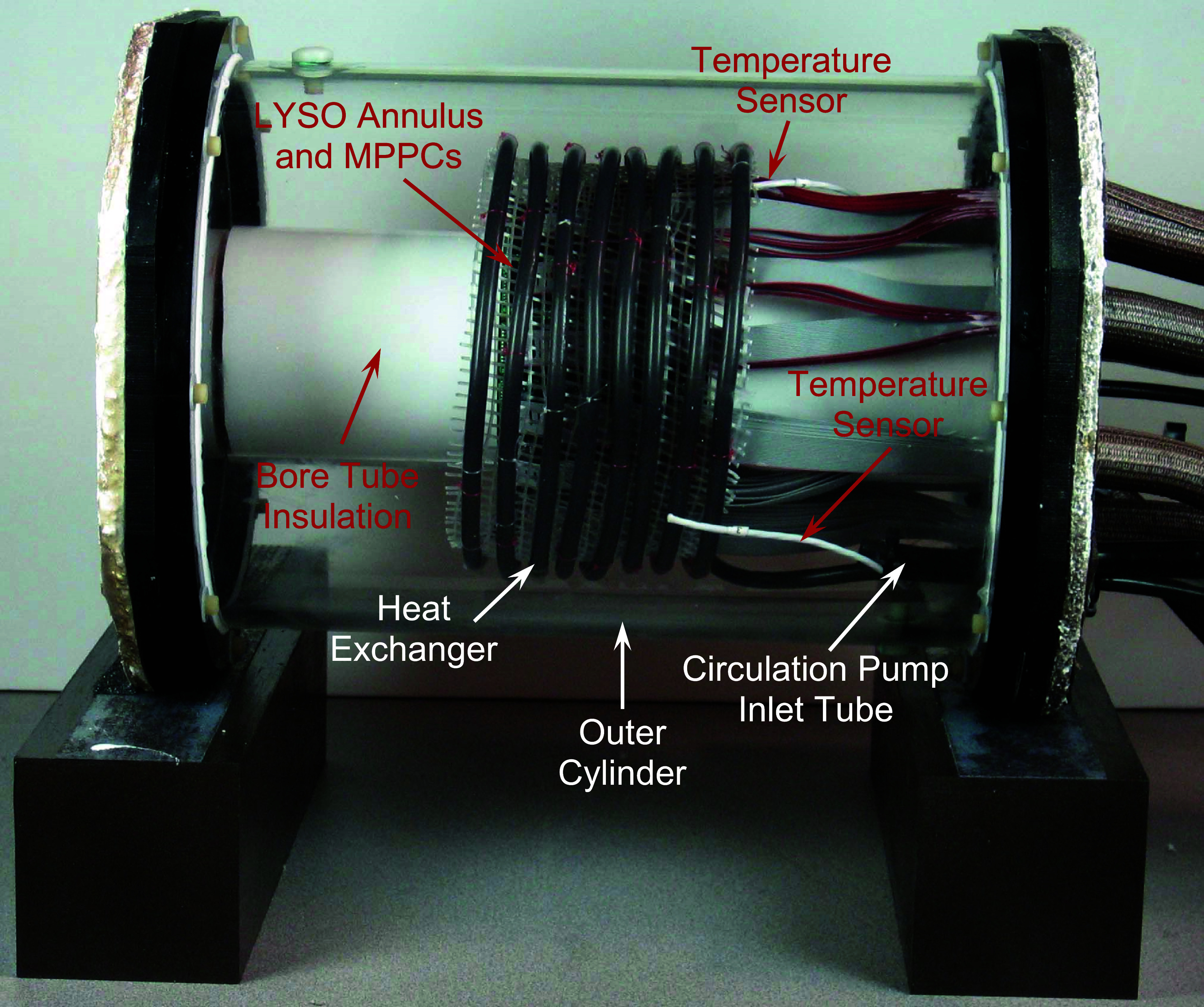
Interior components of the AnnPET gantry enclosed in the outer cylinder that is used to contain the colling fluid.

To enhance performance of the MPPCs, they are immersed in a dielectric fluid (ElectroCool® EC-110 single-phase dielectric coolant (Engineered Fluids, Inc., Tyler, TX)) cooled to ∼12 °C. It requires a one hour cool down period to reach this temperature. During operation, following the initial cool down period, the temperature of the MPPCS remains relatively constant (±0.2 °C). Lowering the temperature of the MPPCs reduces their overvoltage, resulting in an increase in gain (∼33%) and reduction in dark current (66%), for the bias voltage (32 V) used by AnnPET (Stolin *et al* 2013, Raylman and Stolin 2016 and Hamamatsu Photonics 2024). Perhaps, more importantly, immersion in the cooled fluid stabilizes the temperature of the MPPCs, minimizing change of their performance (gain and noise) during scanning. The dielectric fluid was cooled indirectly with a heat exchanger fabricated from thermally conductive tubing (H2^TM^) (Fluorotherm, LLC, Parsippany, NJ) through which a cooled 50/50 mix of distilled water and glycol was pumped. The fluid was cooled and circulated through the heat exchanger with a minichiller (MiniChiller300) (Huber USA, Inc., Raleigh, NC). The temperature of each MPPC array is monitored with a platinum thermistor mounted on the readout electronics board connected to the MPPCs. Temperatures at three additional positions inside the housing (the surface of the heat exchanger, the fluid surrounding the inner surface of the heat exchanger and the fluid surrounding the inner surface of the heat exchanger near the MPPC arrays) are monitored using submersible thermistors (National Instruments, Inc., Austin, TX). The fluid is circulated within the scanner to promote a uniform temperature distribution in the gantry using an externally mounted, self-priming pump (Auhafaly, China). In addition to the electrical connections to the MPPC arrays, connections to the temperature sensors, as well as the tubes carrying cooling fluid to the heat exchanger, and tubes to and from the gantry circulation pump pass into the gantry tank via fluid-tight feedthroughs in the rear endplate. The body of the gantry consists of an acrylic, flanged cylinder (inner diameter = 20.3 cm, wall thickness = 5 mm). Figure 3 shows this cylinder as well as the internal components of the scanner. In the final configuration, the acrylic cylinder and endplates are covered with sheets of aerogel insulation to aid in maintaining the scanner’s operating temperature.

### 2.3. Data acquisition (DAQ)

Each MPPC array is readout using custom front-end electronics (AiT Technologies, LLC, Newport News, VA) that produces sixteen channels of analog data (one channel for each MPPC in the array). We utilized individual instead of multiplexed readouts to maximize the granularity of the measurements of the photon distribution at the expense of an increase in data channels. These signals are transmitted over two, twenty-conductor ribbon cables (eight channels per cable). The cables also convey a single bias voltage to the MPPC array and signals from the onboard temperature sensors. They are connected to an adapter board (AB16-S14160) (AiT Technologies, LLC, Newport News, VA) that combines the two separate sets of eight channels onto a single forty-conductor ribbon cable that carries all sixteen data channels, bias voltage and temperature information. The adapter board also sums the amplitudes of the sixteen channels received from the MPPC array and outputs it on a LEMO^TM^ cable. This sum signal is an indication of the amount of scintillation light (energy) detected by the MPPC array. The sixteen MPPC analog signals on the forty-conductor ribbon cable are routed to an analog-to-digital (ADC)/interface module (SIPM DAQ-16, AiT Technologies, LLC, Newport News, VA). This device serves three purposes. First, it supplies the computer-controlled DC bias voltages transmitted to the MPPC array. Second, it digitizes the sixteen channels of analog data from the array with a 12-bit ADC. And, finally, it processes the temperature data from the platinum resistor on the MPPC readout electronics. Communication with this module, including transmission of the digitized MPPC array data, is performed via a USB2.0 connection to a DAQ computer (rackmount Dell Poweredge R730). Each DAQ computer can control and receive data from eight MPPC arrays, representing the data from two facets, via two four-channel USB 2.0 expansion cards. Therefore, the complete system requires seven DAQ computers to process data from the fifty-six MPPC arrays (14 facets × 4 MPPC arrays per facet). Coordination of DAQ and image reconstruction is performed by a single computer (rackmount Dell Precision 7910) that is networked with the DAQ computers.

**Figure 4.**
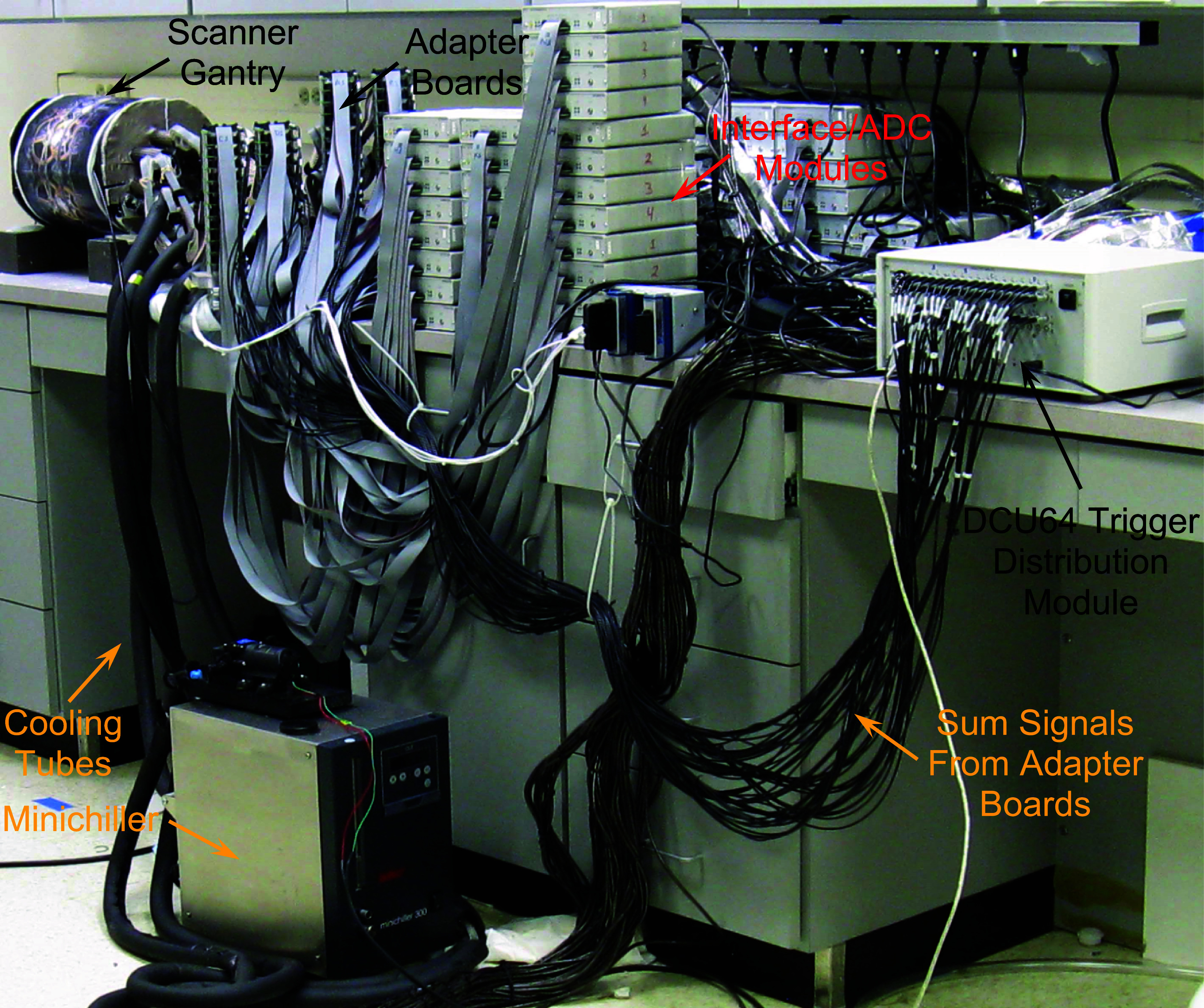
The complete AnnPET system.

Digitization of the analog data from the MPPC arrays is initiated by identification of a coincidence between signals from two facets. To accomplish this task, the fifty-six sum (energy) signals produced by the adapter boards are routed to a trigger distribution module (DCU64) (AiT Technologies, LLC, Newport News, VA) via LEMO^TM^ cables. A voltage threshold of 125 mV is applied to all incoming sums to filter out low level electronic noise. This value was selected as the setting that minimizes single event rate when no radioactivity is present in the scanner without measurably reducing the coincidence count rate when radioactivity is present. The DCU64 first combines the sum signals into fourteen groups of four, each group represents the total amount of scintillation light (energy) detected by the four MPPC arrays mounted on a single facet. These facet-sums are searched to determine if two facets detected coincident annihilation photons (coincidence window is 2 ns), no energy thresholding is applied to these signals. This sized window is intended to reduce the number of random coincidences and is the narrowest we can achieve with our electronics architecture. Each facet is in coincidence with seven opposing ones, which means that the sensitive volume spans the complete volume of the scanner’s bore. If a coincidence is detected, LVTTL trigger signals are sent to all the ADC modules to initiate digitization of the analog signals present at their inputs. The complete AnnPET system is shown in figure 4.

### 2.4. Data processing and image reconstruction

**Figure 5.**
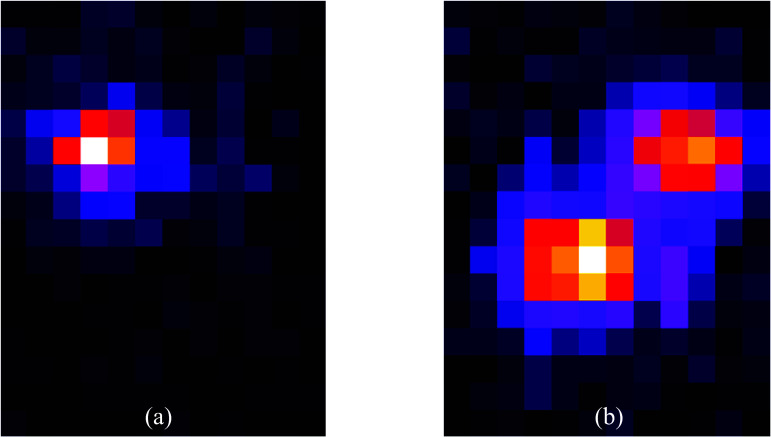
Representative scintillation photon distributions (12 × 16 individual elements) generated by the interaction of an annihilation photon in the scintillator acquired from the actual AnnPET scanner. (a) Optical photon distribution generated by a photoelectric absorption (PE) or Compton scatter (CS) with escape event (PSE) and (b) optical photon distribution generated by a CS-PE event (note that it is not possible to discriminate the CS-generated event from the PE-generated event).

To approach the higher resolution theoretically possible with PET scanners utilizing monolithic scintillator, sophisticated methods to identify annihilation photon interaction points in the scintillator are required (Ling *et al* 2007, Wang *et al* 2013). For AnnPET this task is even more challenging than for a detector using blocks of scintillator given the unique optics present in the faceted annulus. The process begins with identification of which two of the fourteen detector facets are the primary and secondary facets that interacted with the two annihilation photons. To achieve this goal the digitized data is searched to find the facet that contained the maximum signal (defined as the primary facet). The signals from the opposing seven facets are then searched for the facet that contains maximum signal (defined as the secondary facet). Next, the digitized data from facets to each side of the primary and secondary facets are combined with the digitized data from the primary and secondary facets to produce two, 3 × 4 arrays of data from the MPPC arrays. Hence, each of these structures contains 12 × 16 individual elements, since each array is made up of 4 × 4 individual MPPCs. These matrices represent the distributions of detected optical photons produced by the annihilation photon interactions in the scintillator. Figure 5 shows examples of these distributions. Grouping of signals from adjacent facets is necessary because the optical photons produced by annihilation photon interactions in the scintillator can migrate to regions that extend beyond the boundaries of the primary/secondary facets. Our investigations found that >90% of the optical photons from an event are contained within a span of three facets.

To identify the three-dimensional location of an annihilation photon interaction in the annulus that define the endpoints of an LOR, the 12 × 16 photon distributions (figure 5) are processed by a deep residual, convolutional neural network (DR-CNN). Briefly, the network employs ten layers, appropriate for the classification of optical images, which is essentially our task. It is implemented in PyTorch 1.4 and CUDA 10.1, running on an NVIDIA GPU RTX 2080Ti (4352 Cores) (Jaliparthi *et al* 2021). The network was trained using a data set consisting of ∼16 million events created using a GEANT 4 (Agostinelli *et al* 2003) computer simulation of the AnnPET detector (Jaliparthi *et al* 2021). Training set size was estimated from our previous study of the use of this DR-CNN with AnnPET (Jaliparthi *et al* 2021). GEANT4 has been demonstrated to accurately produce optical photon distributions in monolithic blocks of LYSO (van der Laan *et al* 2010, Grahe *et al* 2017, Stockhoff *et al* 2019). Our simulation employed the G4EmStandardPhysics_option4 physics package and the UNIFIED optical photon interaction model (Nilsson *et al* 2015, Khodaei *et al* 2023). For each simulated positron annihilation interaction in the simulated detector, an optical photon distribution, like the ones detected by the scanner hardware (figure 5), was created. The pixel values in each distribution were normalized so that the maximum value is 1. Utilization of a simulated training data eliminates the necessity of performing extensive, imprecise and time-consuming measurements using a collimated source in the actual scanner. The positions of the events provided by the simulation are known with very high accuracy. Several groups have used simulated data to train neural networks designed to position events in monolithic blocks of scintillator (Sanaat and Zaidi 2019, Decuyper *et al* 2021, Carra *et al* 2022, Clement *et al* 2022).

There are three predominant types of interactions that annihilation photons can undergo in the annulus: Photoelectric effect absorption (PE), Compton scatter with scattered photon escape (CSE) and Compton scatter with the scattered photon undergoing subsequent photoelectric absorption (CS-PE). Application of a neural network is necessary to obtain accurate event positioning given these phenomenon and the complex nature of the optics in the annulus. Specifically, scintillation light distributions resulting from PE, CSE and CS-PE interactions can become distorted at the interface between facets and from reflections from the inner surface of the annulus. A previous study found that the DR-CNN method was more accurate in identifying interaction points in the annulus than the center-of-mass method (Jaliparthi *et al* 2021).

Since our DR-CNN was trained using data sets created by GEANT4 but is used to make position predictions from real data acquired by the AnnPET scanner, the real data must be pre-processed prior to analysis by the neural network. This task is necessary because the two sets of data, while similar in that they represent the optical photons detected by arrays of MPPCs, do not possess the same pixel intensity scaling and background. The real data contains background signals from MPPC noise and the presence of intrinsic radioactivity in LYSO not included in the simulation. To reduce background, a small fraction of the sum of pixel values in the 12 × 16 photon distributions (f-factor) is subtracted from each pixel (Weisenberger *et al* 2001). An f-factor of 0.0025 was found to maximize spatial resolution and image uniformity when applied to reconstructed images of a ^22^Na point source and uniform cylinder of ^18^F solution, respectively. The scaling of the two data sets is harmonized by normalizing the pixels in the real optical distribution to a maximum value of 1, recall the simulated distributions are also normalized to a maximum value of 1.

The coordinates of each annihilation photon interaction in the scintillator (*x, y* and *z*) estimated by the DR-CNN lie within the confines of the annulus of scintillator. Our reconstruction software, however, requires that endpoints of LORs reside on discrete, pixelated, flat structures (Smith and Raylman 2006). To transform the initial data into this format, we project each LOR endpoint estimated by the DR-CNN on to one of fourteen virtual, infinitely thin, pseudo-detectors 3 cm from the center of the scanner. Each of these ‘detectors’ is modeled as a 16 × 84 array of 0.84 × 0.84 mm^2^ ‘detector elements’. They are assumed to be perfect absorbers with no DOI effects. Intersected detector elements are identified and defined as the endpoints of the projected LOR and stored in list mode format. The raw data are corrected for photon absorption by application of an attenuation map calculated for the scanned object. The number of random coincidences in the data are estimated by using the single event rates for each MPPC array measured by the DCU64 module (Brasse *et al* 2005). Images are reconstructed from these data using the maximum likelihood expectation maximization algorithm (MLEM). The reconstructed image voxel size is 0.25 mm^3^.

### 2.5. Performance characterization

Basic performance characteristics of AnnPET were assessed using the NEMA NU4-2008 test protocols (NEMA, National Electrical Manufacturers Association 2008). Spatial resolution was measured by imaging a point source of ^22^Na (1.85 MBq) (Eckert & Ziegler USA, Valencia CA) mounted in a 1 cm^3^ block of acrylic positioned at several transaxial locations (5, 10, 15 and 18 mm from the central axis of the scanner) at the center of the scanner’s axial field-of-view (FOV) and at one quarter of the axial FOV from center. Note that 18 mm is the largest radial offset from the center of the scanner possible with this source. At this location, the source is only 1.65 cm from the inner surface of the scintillator. As specified by the NEMA NU4-2008 protocol, the data were reconstructed using the single slice rebinned-filtered backprojection (SSRB-FBP) method. The full-width-at-half-maximum and full-width-at-tenth-maximum (FWHM and FWTM) of the intensity profiles were measured from these images.

Detection sensitivity was measured by placing the ^22^Na point source at fifteen positions separated by 5 mm along the central axis of the scanner. These data were used to calculate detection sensitivity at each location. Count rate performance was evaluated using the NEMA NU4-2008 mouse-like phantom containing ∼16 MBq of ^18^F at start of the measurements. Eighteen measurements were obtained over approximately ten hours. From these data, true event rate, system random rate, system scattered rate, system noise equivalent count rate (NECR) and total system event rate were measured as the ^18^F decayed. Finally, the NEMA NU4-2008 image quality (IQ) phantom (filled with 2.96 MBq of ^18^F) was scanned to assess image uniformity, recovery coefficient (RC) for the phantom’s hot rods ranging in diameter from 5 mm to 1 mm and accuracy of the image corrections (spillover ratio (SOR) for cylinders filled air and water). Unlike the images utilized to measure spatial resolution and in accordance with the NEMA NU4-2008 protocol, the data from the IQ phantom were reconstructed using the MLEM algorithm, with corrections for photon attenuation and random coincidences, as described above.

## 3. Results

**Figure 6.**
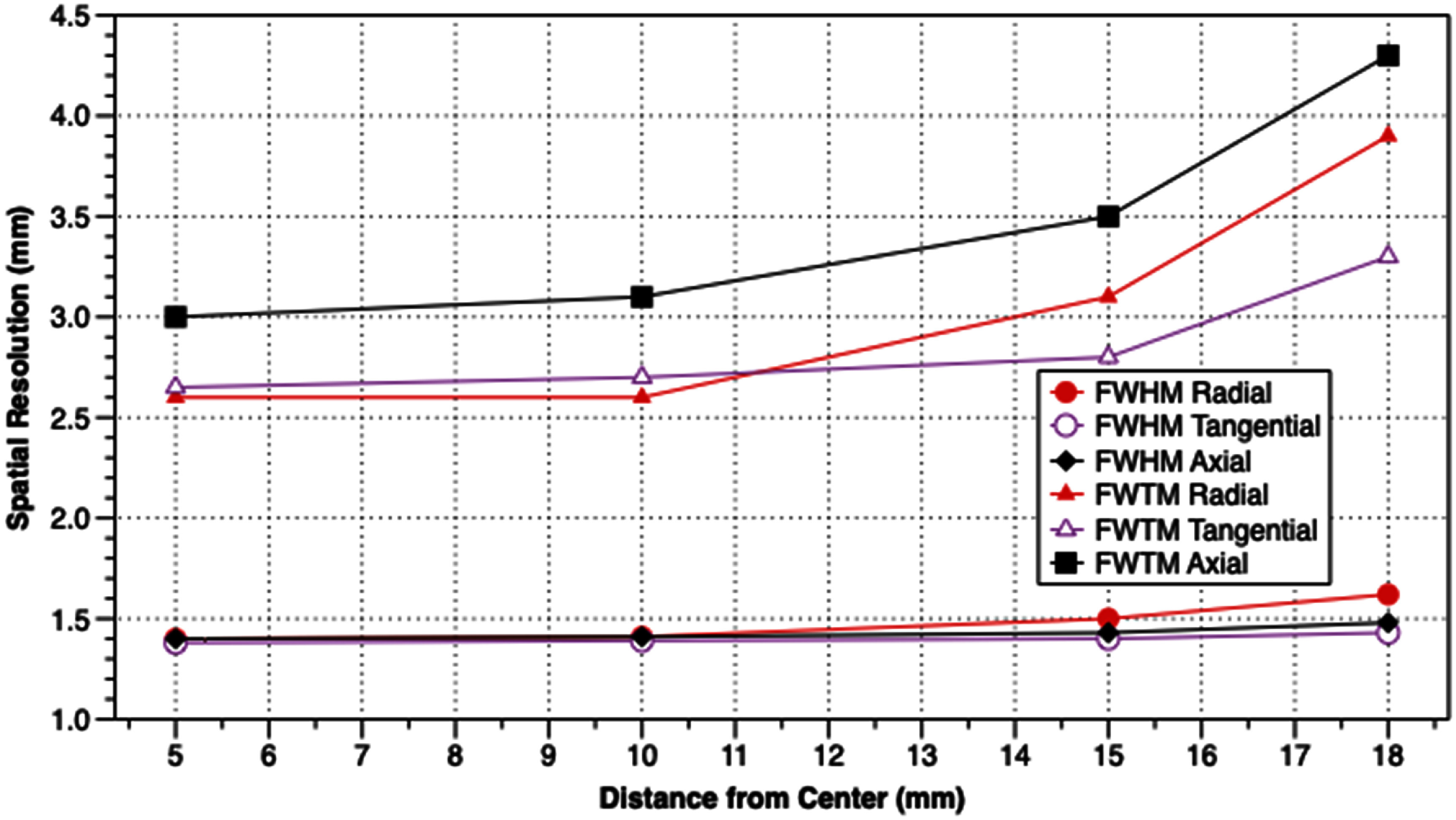
Plots of spatial resolution (FWHM and FWTM) measured at the axial center of the scanner.

**Figure 7.**
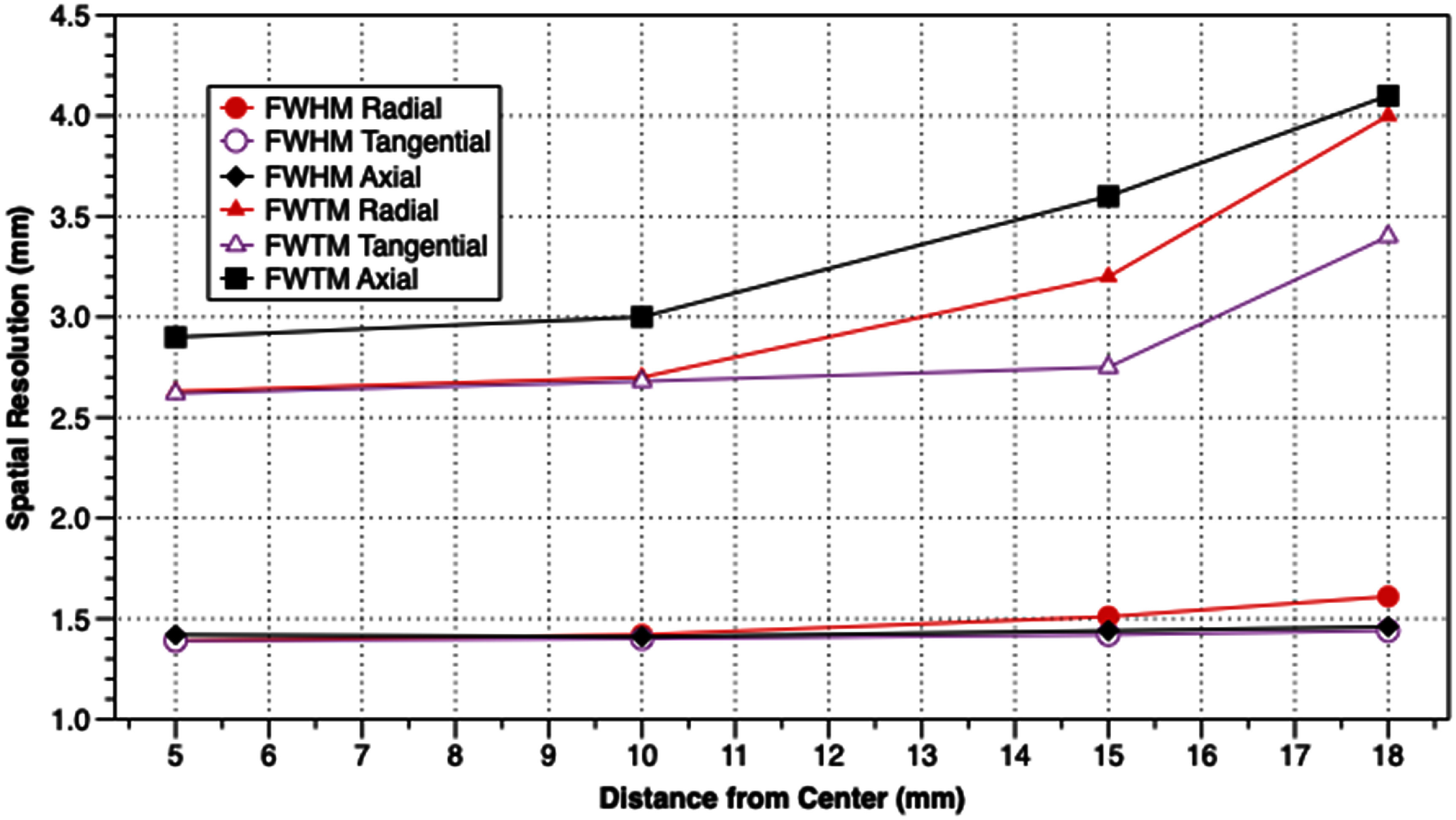
Plots of spatial resolution (FWHM and FWTM) measured at one quarter of the axial FOV (1.8 cm) from the center of the scanner.

**Figure 8.**
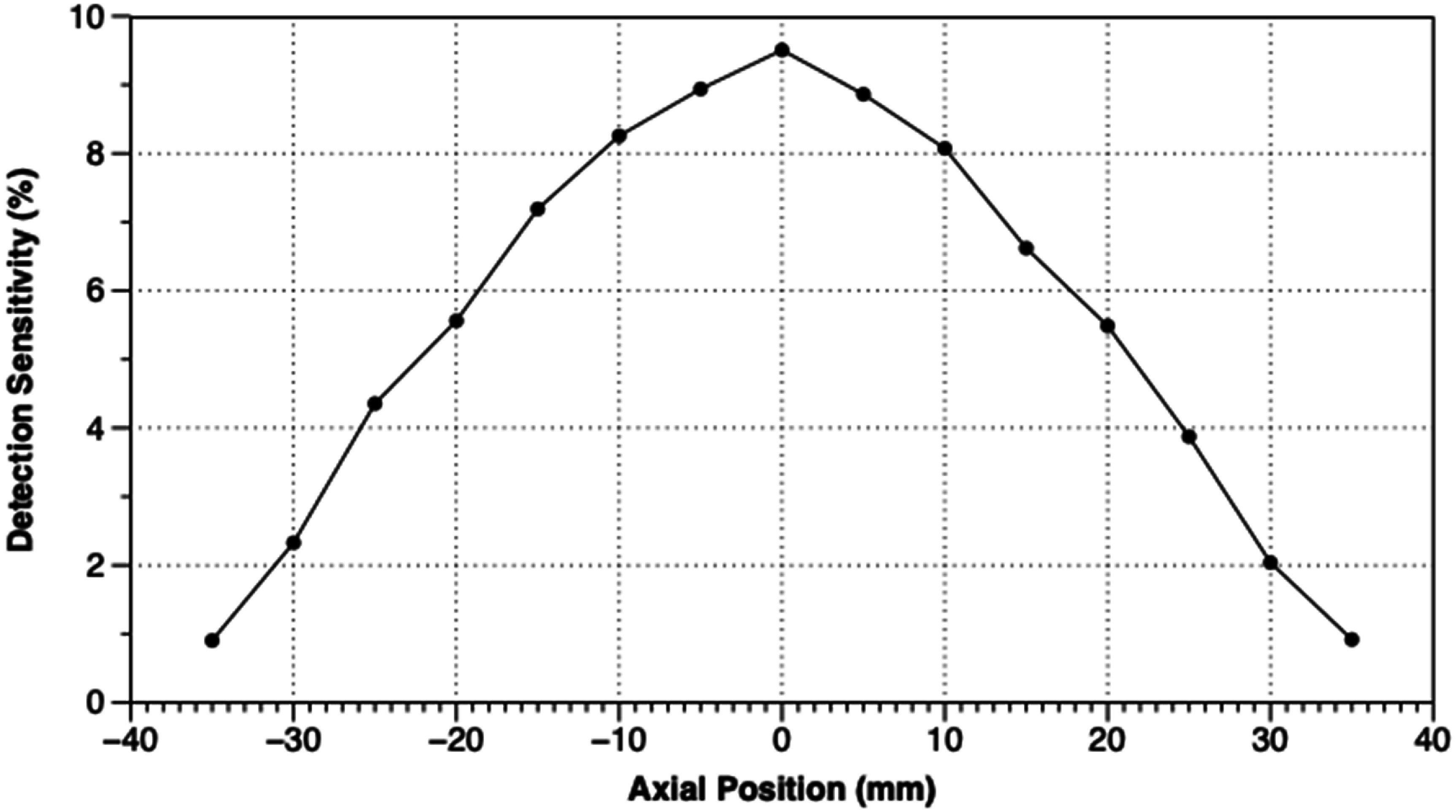
Axial profile of sensitivity.

**Figure 9.**
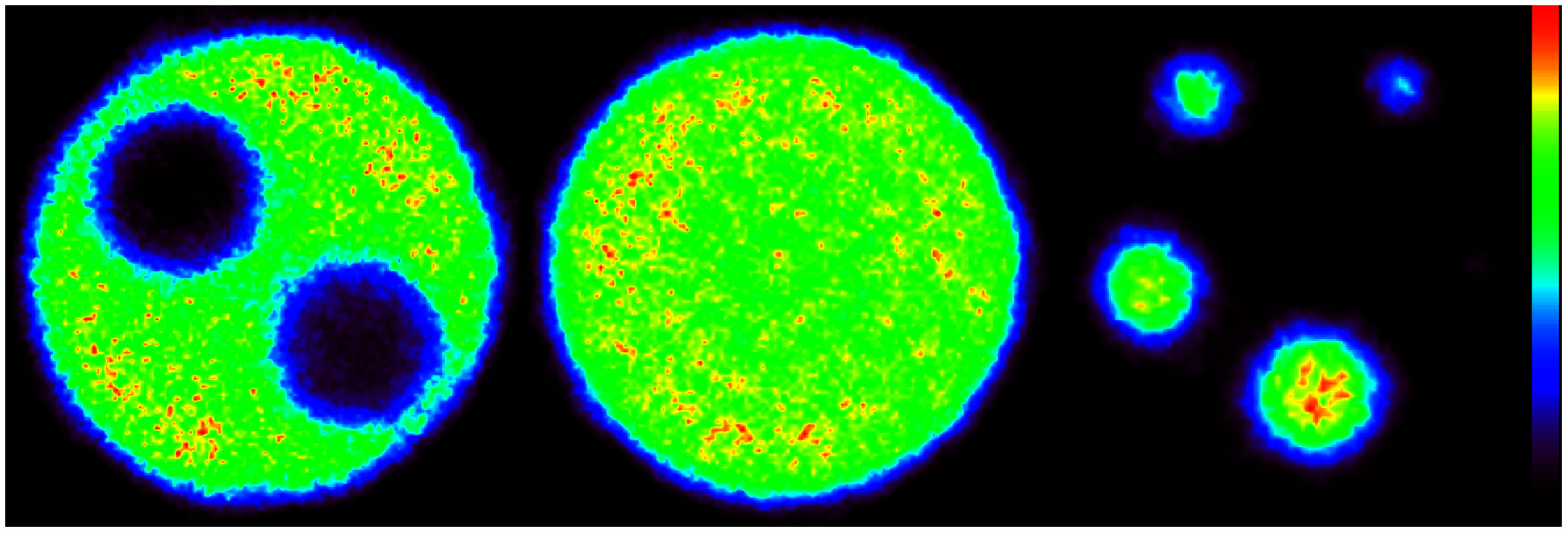
Representative images of the spillover ratio, uniformity and recovery coefficient sections of the IQ phantom.

**Figure 10.**
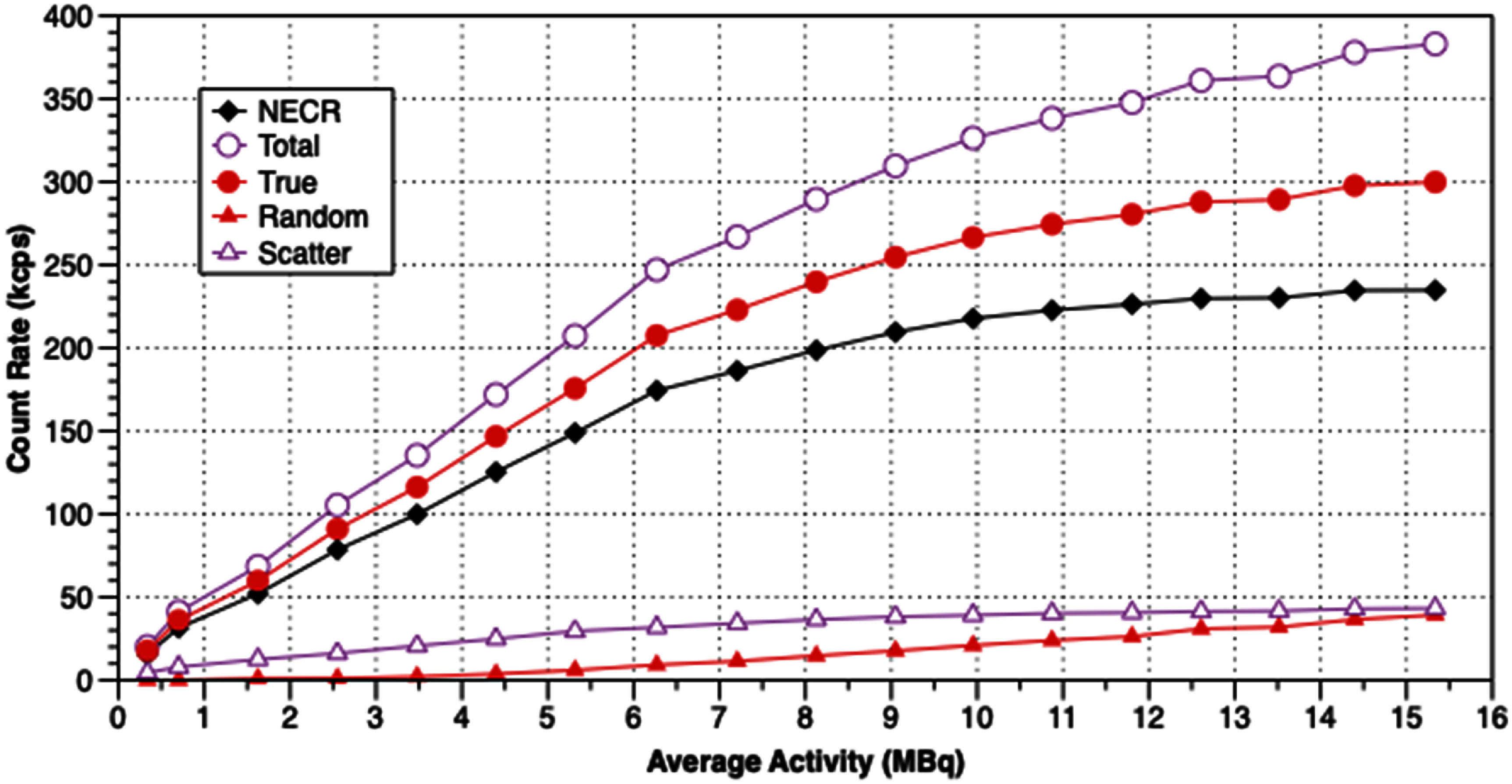
Plots showing the results from the count rate performance measurements with the NEMA NU4-2008 mouse like phantom.

Results from measurements of spatial resolution, FWHM and FWTM of the images of the ^22^Na point source at various positions in the scanner, are shown in table 1, and in figures 6 and 7. Peak detection sensitivity is 9.5% (0.086 cps Bq^−1^), the integrated absolute detection sensitivity is 82.9% and the integrated detection sensitivity is 0.751 cps Bq^−1^. The axial profile of AnnPET’s detection sensitivity is shown in figure 8. Results from the imaging of the IQ phantom are summarized in tables 2–4. Figure 9 shows representative images of this phantom. System scatter fraction is 13.1%. Finally, figure 10 shows the results of the count rate performance measurements. The peak NECR is 234 kcps at 14.4 MBq (418.5 MBq ml^−1^).

**Table 1.** Spatial resolution values (mm).

Reconstructed image pixel size (mm): 0.25								

Slice thickness (mm): 0.25								

At axial center								

	5 mm		10 mm		15 mm		18 mm	

	FWHM	FWTM	FWHM	FWTM	FWHM	FWTM	FWHM	FWTM

Radial	1.40	2.60	1.41	2.60	1.50	3.10	1.62	3.90
Tangential	1.38	2.65	1.39	2.70	1.40	2.80	1.43	3.30
Axial	1.40	3.00	1.41	3.10	1.43	3.50	1.48	4.30

At 1/4 axial FOV from center								

Radial	1.39	2.63	1.42	2.70	1.51	3.20	1.61	4.00
Tangential	1.39	2.62	1.40	2.68	1.42	2.75	1.44	3.40
Axial	1.42	2.90	1.41	3.00	1.44	3.60	1.46	4.10

**Table 2.** Report for uniformity test (arbitrary units).

	Mean	Maximum	Minimum	%STD
Uniformity	52.11	64.86	40.52	3.32

**Table 3.** Report for recovery coefficient test.

Rod diameter	1 mm	%STD	2 mm	%STD	3 mm	%STD	4 mm	%STD	5 mm	%STD
RC	0.08	32.3	0.3	33.8	0.46	27.6	0.7	27.7	0.71	31.4

**Table 4.** Report for Accuracy of Corrections.

Region	SOR	%STD
Water-filled cylinder	0.06	11.1
Air-filled cylinder	0.04	12.6

## 4. Discussion

As pre-clinical PET systems are becoming increasingly integral parts of research programs, continued investigations of potentially enhanced methods for efficiently producing high resolution images are necessary. In this study we investigated the performance characteristics of a monolithic, annular tetradecagonal scintillator-based pre-clinical PET scanner constructed by our group. The goal is to produce a system with high detection sensitivity with high, uniform, spatial resolution that is suitable for integration with an EPRI system. The results shown in table 1 and the plots shown in figures 6 and 7 demonstrate the good spatial resolution of the system. Notably, the uniformity of spatial resolution as a function of position is particularly good. For example, radial resolution is degraded by ∼16% when the point source is moved from 5 mm from center to 18 mm from center (1.65 cm from the surface of the scintillator), illustrating AnnPET’s resistance to the effects of DOI. In the tangential and axial dimensions, spatial resolution degrades by only 3.6% and 5.7%, respectively. The relatively small degradation in spatial resolution in all three dimensions as the source was moved from 5 mm from center to 18 mm is due to the use of continuous scintillator in conjunction with a neural network to determine interaction points, facilitating accurate localization of annihilation interaction points in the scintillator.

While the spatial results obtained with our system are good, there are some effects that limit performance. Perhaps the most likely source of reduced spatial resolution is the situation where a Compton scatter event is followed by absorption of the scattered photon in a photoelectric interaction (CS-PE event), resulting in a photon distribution with two discrete peaks (figure 5(b)). When this distribution is presented to the DR-CNN, there is ambiguity as to which of these peaks was produced by the first interaction (the Compton scatter event), which is the position that should be identified as the LOR endpoint. If the network identifies the photoelectric effect-related peak instead of the Compton scattering-related peak as the primary interaction point, then the endpoint(s) of the LOR(s) is(are) displaced, compromising spatial resolution.

Note that our measurements of spatial resolution are lower than those predicted from our simulations of the system (Jaliparthi *et al* 2021). Specifically, our simulations predicted FWHMs of 0.8 mm, 0.7 mm and 0.71 mm in the radial, tangential and axial dimensions, respectively, at 5 mm from scanner center. At 15 mm from the center, these numbers change slightly to 0.83 mm, 0.72 mm and 0.5 mm. The differences between these results and those measured in this study are most likely to due to small inaccuracies in the data harmonization process.

AnnPET’s spatial resolution results compare well with commercially available pre-clinical PET scanners that utilize rings of discrete, monolithic blocks of scintillator. For example, Pajak *et al* reported that for the two ring Bruker Albira system, the FWHM SSRB-FBP of images of a ^22^Na point source 5 mm from center is 1.92 mm, 1.31 mm and 2.59 mm in the radial, tangential and axial dimensions, respectively (Pajak *et al* 2016). At 15 mm from center, these values are: 5.14 mm, 1.14 mm and 2.59 mm. For the MOLECUBES *β*-CUBE PET scanner, Krishnamoorthy *et al* reported a radial FWHM of ∼0.95 mm at 5 mm from scanner center and ∼0.97 mm at 15 mm from center (2018). Individual values for tangential and axial resolution were not reported. This system utilizes a ML clustering algorithm to estimate annihilation interaction points in the scintillator, in contrast to the application of a DR-CNN in AnnPET, which may be the reason for the very good resolution reported for *β*-CUBE ‘s radial spatial resolution.

Comparisons of AnnPET’s spatial resolution measurements to those reported by Xu *et al* (Huazhong University of Science and Technology) (2019) and Freire *et al* (i3M) (2022) for PET scanners based on single monolithic annuli of scintillator are challenging because neither group used the NEMA NU4-2008 spatial resolution protocol. Specifically, Xu *et al* reported the accuracy in identifying the circumferential and axial positions of photon interactions in their cylindrical detector to be 0.94 mm and 2.45 mm, respectively, not spatial resolution (2019). Freire *et al* reported an average FWHM of 1.4 mm and 1.3 mm in the radial and axial dimensions, respectively (2022). Tangential resolution was not reported because they used a non-standard method for measuring resolution. Specifically, they performed their measurements on sinograms created from processed data rather than reconstructed images of a point source.

Detection sensitivity for AnnPET compares well with scanners constructed from rings of multiple discrete detectors based on monolithic scintillator. For example, the peak detection sensitivity for the Albira scanner was reported to be 5.29% (Pajak *et al* 2016), the peak detection sensitivity for the *β*-CUBE scanner was reported to be 12.4% (Krishnamoorthy *et al* 2018), both results compare well with the 9.5% measured for AnnPET. The higher sensitivity for *β*-CUBE compared to AnnPET is likely related to the fact that it is 80% longer than AnnPET (13 cm for *β*-CUBE compared to 7.2 cm for AnnPET). The group from Huazhong University of Science and Technology did not report detection sensitivity measurements, the i3M group reported a peak sensitivity of 3.8% (Freire *et al* 2022), which may be due primarily to the fact that they only populated approximately one-third of their scintillator with SiPMs. AnnPET’s good detection sensitivity is due to its small bore, lack of gaps between detector modules and the fact that we do not reject Compton scattering events that occur in the scintillator.

Images of the NEMA NU4-2008 IQ phantom (figure 9) were used to assess the imaging capabilities of the scanner. The overall good quality of these images demonstrates that the use of simulated data was successful in accurately training the DR-CNN. Results from analyses of these images indicate higher image uniformity compared to the *β*-CUBE scanner (Krishnamoorthy *et al* 2018) (3.32% STD versus 7.43% STD). Image uniformity was not reported for the Albira scanner. Neither the Huazhong University of Science and Technology group nor the i3M group reported results for image uniformity. To demonstrate how spatial resolution affects the scanner’s capability for quantifying the amount of activity in small objects, which is affected by the partial volume effect, RC from images of the phantom’s hot rod section were measured. For the most challenging two smallest rods (1 mm and 2 mm diameter), RC of 0.08 and 0.30, respectively, were measured. In comparison, values of 0.05 and 0.30, respectively, were reported for the Albira scanner (Pajak *et al* 2016) and 0.29 and 0.67 for the *β*-CUBE scanner (Krishnamoorthy *et al* 2018). The very good results for *β*-CUBE are a consequence of its high spatial resolution. Neither of the groups that constructed an annular scanner reported RC from images of this phantom. The SOR for the Albira scanner was reported to be 0.22 and 0.14 for the water-filled and air-filled cylinders, respectively (Pajak *et al* 2016). SORs for the *β*-CUBE scanner were reported to be 0.08 for both cylinders (Krishnamoorthy *et al* 2018), which is slightly poorer compared with the 0.06 and 0.04 measured from AnnPET images of the water and air-filled cylinders. AnnPET’s relatively low SORs are likely due to the small diameter of AnnPET’s bore, limiting displacement of scattered photons (Adler *et al* 2022).

Finally, the count rate measurement results (figure 10) illustrate the efficient DAQ system employed in AnnPET. For the mouse like phantom, its peak NECR (234 kcps at 14.4 MBq) compares well with that reported for the Albira scanner (72 kcps at 71 MBq) (Pajak *et al* 2016) and the *β*-CUBE scanner (300 kcps at 33.3 MBq) (Krishnamoorthy *et al* 2018). For the continuous tube scanner fabricated by the Huazhong University of Science and Technology group, the reported peak NECR is 16.9 kcps at 12.7 MBq (Xu *et al* 2019). The i3M group reported a peak NECR 40.6 kcps at 9.99 MBq for their faceted-annulus scanner (Freire *et al* 2022).

## 5. Conclusions

The AnnPET scanner is designed to leverage the potential performance benefits of PET systems based on detectors utilizing a single, annular piece of scintillator, which include high resolution, due to the ability to accurately estimate DOI, and high detection sensitivity, due to the lack of gaps between detector modules. Use of a single monolithic piece of scintillator simplifies construction compared to those that use rings of discrete detectors (no support structure for the modules is required, for example). The results from this investigation demonstrate that this design, including use of immersion cooling and a DR-CNN, show promise to produce a pre-clinical imaging system with very good imaging capabilities. They also demonstrate that DR-CNNs used for event position can be trained successfully with GEANT4-produced data.

While AnnPET’s performance compares well with other pre-clinical PET scanners based on continuous scintillator, there are opportunities for improvement. This effort will center around improving the accuracy in determining annihilation photon interaction points in the scintillator, which will enhance spatial resolution. Specifically, we are planning to explore ways to remove the ambiguity encountered by the neural network when presented with a bi-peak photon distribution (CS-PE event) (figure 7(b)), which introduces error in the estimations LOR endpoints. We plan to study the use of a model-based secondary analysis algorithm to obtain better identify of the coordinates of the Compton scatter event and/or modification of the neural network to identify the CS-PE events so that they can be excluded from the reconstructed data. We are also investigating use of a reconstruction algorithm that does not require re-formatting of the data from the DR-CNN to pixelated data structures. Additionally, we are in the process of adapting a single scatter model (Ollinger 1996) to correct AnnPET images for Compton scattered annihilation photons in the object. Finally, the AnnPET scanner will ultimately be combined with an EPR scanner to explore the complementary roles of glucose metabolism and hypoxia in cancer.

## Data Availability

The data cannot be made publicly available upon publication because they are not available in a format that is sufficiently accessible or reusable by other researchers. The data that support the findings of this study are available upon reasonable request from the authors.
